# Analysis of the effect of integrons on drug-resistant *Staphylococcus aureus* by multiplex PCR detection

**DOI:** 10.3892/mmr.2013.1284

**Published:** 2013-01-21

**Authors:** CHUNFENG REN, YONGJING ZHAO, YAN SHEN

**Affiliations:** 1 Department of Clinical Laboratory, The First Affiliated Hospital of Zhengzhou University, Zhengzhou, Henan 450052, P.R. China; 2 Zhengzhou Children’s Hospital, Zhengzhou, Henan 450053, P.R. China

**Keywords:** integron, multiplex PCR, multidrug resistance, *Staphylococcus aureus*

## Abstract

The aim of this study was to detect class I, II and III integrons using multiplex PCR, and to analyze the role that these integrons play in mediating multidrug-resistant *Staphylococcus aureus* (SA). The sensitivity of SA to 20 types of antibiotics was examined using the K-B method. A genomic DNA extraction kit was used for extracting genomic DNA and a high-purity 96 plasmid extraction kit was used for extracting plasmid DNA. Class I, II and III integrons were amplified using multiplex PCR. Agarose gel electrophoresis was used for analysing amplification products. The positive rate of class I and II integrons in the plasmid DNA from SA was higher compared to that of the genomic DNA. The positive rate of class I integrons was highest in the group with multidrug resistance to amoxicillin/clavulanic acid, piperacillin/tazobactam, ciprofloxacin, tetracycline, rifampin, imipenem, cefazolin, cefuroxime, levofloxacin and gentamicin. As regards integron detection in the plasmids from drug-resistant SA strians obtained from sputum, blood, cerebrospinal fluid, drainage fluid, excretion and urine specimens, the difference in the detection rate of class I integrons among the six types of specimens was significant. Multiplex PCR is an effective method to detect class I, II and III integrons. The SA plasmid is the main carrier transferring integrons. Integrons mediate the formation of SA multidrug resistance.

## Introduction

The drug resistance mechanisms in bacteria, which involve the production of inactivation enzymes, the alteration of target protein synthesis and the alteration of membrane permeability, thus reducing the aggregation antibiotics and producing biofilms, have become hot research topics (1). In 1989, Stokes and Hall first put forward the concept of integrons (2). Integrons are a hereditary unit for gene capture and expression, situated in the bacterial plasmid, chromosome or transposon, which have the capability of site-specific recombination. They can also selectively capture or remove various specific drug resistance box genes, and transfer their drug resistance genes to different strains or different bacterial genera through functions, such as transformation, transduction and conjugation, a mechanism which accelerated the spread and dissemination of bacterial drug resistance (3). New integron types are continuously being discovered and the number of identified integron types has increased. However, research remains focused on class I, II and III integrons. Due to the widespread integration effect of the integron integrase gene on bacterial drug resistance and its transitivity among different genetic materials, multidrug-resistant bacteria are capable of being rapidly transferred. From the related literature, we can see that integrons are important in the dissemination of the majority of bacterial multidrug resistance (4).

In China and the rest of the world, the most commonly applied integron detection method is to use the specific primer of various integrons for single PCR and examine them separately (5). It has been reported that integrase gene classification may be achieved by combining restriction enzyme digestion and the PCR method (6), which uses degenerate primers through the integron sequence of class I, II and III. Although this method simplifies the previous method to a certain degree, the classification results are obtained by two reactions and two electrophoresis experiments, which is more time-consuming and uses more materials. Therefore, it is important to improve these methods and establish a rapid detection method. Based on the above conditions, in this study, integrase-specific primers for class I, II and III integrons were designed in order to establish a multiplex PCR method that is capable of detecting the three classes of integrons concurrently.

In recent years, although integrons have been found in Gram-positive bacteria, the role that integrons play in drug-resistant *Staphylococcus aureus* (SA) remains unclear (7). Therefore, SA, the most representative genus of Gram-positive bacteria, was selected to be the subject of this study. The integron carrying conditions of various drug-resistant SA strains, drug-resistant SA strains from different sources, SA plasmid and genomic DNA were analysed using this multiplex PCR method, in order to determine the role that integrons play in mediating multidrug-resistant bacteria.

## Materials and methods

### Materials

#### Strains

All 180 SA strains were collected from clinical specimens from January 1, 2009, to December 31, 2009, including 26 blood specimens, 86 sputum specimens (including throat swab), 15 drainage fluid specimens (including pleural fluid, ascites, dialysate, catheter drain and synovial fluid), 3 cerebrospinal fluid specimens, 32 urine specimens and 18 secretion specimens (including liquor puris). The inoculation, culture and staining, separation culture, study of biochemical events, susceptibility tests, assessment by the Bactest microorganism analytical system and the reported results of the specimens all strictly adhered to the basic microorganism operating regulations. Drug-sensitive slips were purchased from Oxoid Co. (Basingstoke, UK). The purified strains were stored at −80°C. The quality control bacterial strain was SA ATCC25923.

#### Antibacterial agents

Benzylpenicillin, cefoxitin, oxacillin, erythromycin, clindamycin, azithromycin, bactrim, vancomycin, linezolid, amoxicillin/clavulanic acid, piperacillin/tazobactam, ciprofloxacin, tetracycline, rifampicin, imipenem, cefazolin, cefuroxime, levofloxacin, gentamicin and teicoplanin were used. The sensitivity of SA to 20 types of antibiotics was examined using the K-B method. The susceptibility test referred to the CLSI2009 antibacterial susceptibility testing standard.

#### Main reagents

Tris saturated phenol was produced by Shanghai Generay Biotech Co., Ltd. (China), protease K was produced by Amresco Co. (Solon, OH, USA), trichloromethane was purchased from Tianjin Northern Tianyi Chemical Agent Manufacturers (China), drug-sensitive slips were produced by Oxoid Co. and blood agar plates were provided by Beiruite Bio-technology (Zhengzhou) Co., Ltd. (China). The bacterial genomic DNA extraction kit was provided by Beijing Tianjin Biochemical Technology Co., Ltd (China). The high-purity 96 plasmid extraction kit was provided by the Beijing Kangweishiji Biotech Co., Ltd. (Beijing, China). Agarose was provided by the Shanghai Bioengineering Co., Ltd. (Shanghai, China). Primers were synthesised by the Shanghai Bioengineering Co., Ltd. and the sequences were as follows: Class I integron upstream primer, 5′-CCT CCC GCA CGA TGA TC-3′ and downstream primer, 5′-TCC ACG CAT CGT CAG GC-3′; class II integron upstream primer, 5′-GTA GCA AAC GAG TGA CGA AAT G-3′ and downstream primer, 5′-CAC GGA TAT GCG ACA AAA AGG T-3′; class III integron upstream primer, 5′-GCC TCC GGC AGC GAC TTT CAG-3′ and downstream primer, 5′-ACG GAT CTG CCA AAC CTG ACT-3′.

#### Main instruments

The PTC-1148 PCR amplification system was obtained from Bio-Rad (Hercules, CA, USA), and DY-A electrophoretic apparatus was from Shanghai Kangda Electronic Apparatus Manufacturers. The Gel Logic 200 gel imaging system was obtained from the Carestream Health Inc., (NY, USA).

#### Data processing

SPSS13.0 software was used for the statistical analysis of the original data. The Chi-square test was adopted for the rate comparison. Size of test, α=0.05.

### Methods

#### Experimental strains for the in vitro bacteria drug sensitivity test

The inoculation, culture and staining of specimens, separation culture, study of biochemical events, susceptibility tests, assessment by Bactest microorganism analytical system and reported results all strictly adhered to the basic microorganism operating regulations. The judgement of the results was performed according to the CLSI standards, 2009.

#### Strain recovery and enrichment

The purified bacterial specimen was removed from storage (at −80°C). The bacterial specimen was inoculated on the blood agar culture plate using an inoculating loop, then the culture plate was cultured in the 35°C electric-heated thermostatic water bath for 24 h, and this operation was repeated until a single colony appeared. The single colony was then cultured for 24 h in a 35°C electric-heated thermostatic water bath.

#### The extraction of SA plasmid DNA and SA genomic DNA

The high-purity 96 plasmid extraction kit from Beijing Kangweishiji Biotech Co., Ltd. was used for the SA plasmid DNA extraction. The TIANamp Bacteria DNA kit from Beijing Tianjin Biochemical Technology Co, Ltd. was used for SA genomic DNA extraction. The kits were operated according to the manufacturer’s instructions.

#### PCR amplification and product analysis

The reaction conditions for PCR amplification were as follows: Pre-degeneration for 4 min at 94°C, degeneration for 45 sec at 94°C, annealing for 45 sec at 55°C, elongation for 55 sec at 72°C and finally, after 30 cycles, elongation for another 8 min. The amplification products were stored at −20°C.

In order to efficiently screen different types of integrons, the multiplex PCR method was used for integrase gene detection, which saved time and money. The designed primers for the class I, II and III integrons had the following features: Similar PCR reaction annealing temperatures, largely varying products and electrophoresis strips which were easy to differentiate. The laboratory procedure was as follows: Primers of the class I, II and III integrons were set in the same reaction system by the gradient PCR method. The six annealing temperature gradients were between 51–60°C. Finally, the annealing temperature of multiplex PCR was set as 55°C.

After PCR amplification, 5 μl of products were added to the well containing 2% agarose gel (ethidium bromide staining). Products were electrophoresed for 30 min under 100 V with 0.5× TBE liquid as the electrophoretic liquid. Strips were observed using the Kodak gel imaging system.

## Results

### Class I, II and III integrons detected by the PCR method

**Figure 1:**
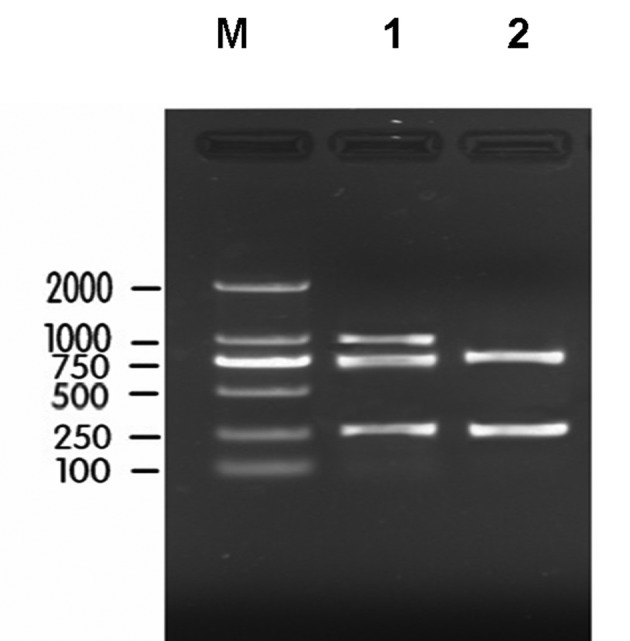
Electropherogram of PCR amplification products of the class I, II and III integrons. M, DNA marker; lanes 1 and 2, multiplex PCR amplification. PCR bands show that the amplification products of class I, II and III integrons were 280, 788 and 979 bp, respectively.

With the primers designed according to the integrase sequence of class I, II and III integrons, the multiplex PCR method was used for the amplification of SA plasmid DNA and SA genomic DNA. Subsequently, 2% agar gel electrophoresis was used to observe the amplification products. The gel electrophoresis found that bands appeared at 280, 788 and 979 bp, and the segment length corresponded with the anticipated results (Fig. 1).

### Drug resistance analysis of SA

The difference in SA drug resistance rates to various types of antibacterial agents was statistically significant (P<0.05). Among the 20 detected antibiotics, the three antibiotics with the highest drug resistance rate in SA were benzylpenicillin (93.7%), erythromycin (81.1%) and azithromycin (79.4%; Table I). The three antibiotics with the lowest drug resistance rate were vancomycin (0%), teicoplanin (2.2%) and linezolid (2.8%). SA exhibited features of multiple drug resistance.

### Role of integrons in mediating drug resistance in SA

#### Comparison of class I and II integron detection in SA plasmids and genomic DNA

The positive rate of class I and II integrons in plasmid DNA was higher than that of genomic DNA, and the positive rate difference was statistically significant (P<0.05) (Tables II and III).

#### Class I and II integron detection in the SA plasmids with different drug resistance

According to the drug selection standards for SA (CLSI 2009) and the different antibiotic-resistant strains of SA, the strains were divided into the following three groups: Group A, single drug resistance strains; group B and C were multidrug resistance strains with different degrees of resistance. The difference between class I integron detection rates in these groups was statistically significant (P<0.01). The class I integron detection rate in group C was higher than that in group A (Table IV). The difference in the class II integron detection rate was not statistically significant (P<0.05). Class I, II and III integrons were not detected in the quality-control strain, ATCC25923.

#### Class I and II integron detection in the SA plasmid DNA obtained from different specimens

The specimens were divided into six groups according to their source (sputum, blood, cerebrospinal fluid, drainage fluid, excretion and urine). The detection rate of class I integrons in the plasmid DNA from the six sources was statistically different (P<0.01). Multiple comparison analyses were then performed on the samples and the difference was found to be statistically significant (P<0.05). The detection rate of class I integrons was the highest in the plasmid DNA from the urine specimen, followed by the sputum specimen, while the detection rate of class I integrons was the lowest in the plasmid DNA from the blood specimen. The detection rate of class II integrons in the plasmid DNA obtained from the six types of specimens was not statistically different (P>0.05) (Table V and VI).

## Discussion

The collected specimens underwent a susceptibility test to 20 types of antibiotics. The results indicated that SA had different degrees of drug resistance to 19 types of antibiotics, with the exception of vancomycin. The top three antibiotics to which SA had the highest drug resistance rate were benzylpenicillin (93.7%), erythromycin (81.1%) and azithromycin (79.4%). SA showed multidrug resistance features. This indicates that the phenomena of drug resistance in SA is currently a serious one.

The bacterial drug resistance situation is becoming increasingly serious, as the production speed of antibacterial agents is unable to catch up with the bacterial drug resistance production speed. Soon after the application of a neotype antibacterial agent, the bacteria that are capable of resisting this type of agent would appear. The generation and transmission mechanisms of the drug resistance genes have become a hot research topic in order to control the spread of drug-resistant bacteria. Integron, the fluid element of genetic transmission, has drawn great attention in mediating bacterial drug resistance (8).

At present, nine classes of integrons may be retrieved from GenBank. However, only the first four classes have been confirmed. Among them, class I integrons have been found more frequently in various types of bacteria, particularly Gram-negative bacteria, which may be observed in a number of studies (9,10). The classification of different integrons is mainly based on differences in the gene structure of integrases (11). The typical integron structure includes 5′ and 3′ conservative regions and a variable gene cluster in the middle. The 5′ conservative end consists of an encoding integrase gene, intI, a recombination site, attl, and a gene segment in the promoter sequence area, which is the basic structure of all integrons. Although several genes carry promoter sequences, the majority of box genes have no promoter sequence; therefore, the 5′ promoter sequence plays an important role in promoting the transcription of its downstream box genes (12). Not all integrons have the 3′ conservative fragment structure. Most class I integrons have a 3′ conservative end, which is comprised of the qacE1 gene which encodes drug resistance in bacteria, the sun gene which encodes drug resistance against sulfamido and an open reading frame of unknown function (4,13). There are a few class I integrons that have no 3′ conservative fragments or lack a typical 3′ conservative sequence (14). Class II integrons are generally situated in Tn7, whose integrase gene, intI2, is internally separated by the terminal codons, becoming the defective gene. The 3′ end of class II integrons is constituted by the gene participating in the Th7 transposition mechanism, which is different from the 3′ conservative end of class I integrons (15). There are only a few reports on class III integrons with the first case found in *Serratia marcescens*(16). Another case was found in *Klebsiella pneumoniae*(15) with two promoter sequences carrying two types of drug resistance box genes, laGEs and blaox/aaIb. Class IV integrons, the so-called super integrons, mainly exist in the *Vibrio cholerae*, according to previous reports (13,17). Class IV integrons carry tens to hundreds of box genes and have several virulence genes, with the exception of a drug resistance gene.

Comparatively, since SA has not been extensively studied, it was selected as the research subject in our study. Only one case with class III integrons was detected; the carrying conditions of class I and II integrons in SA were mainly analysed. According to the known data, the integrase gene, intI1, of class I integrons encodes 337 amino acids (18), and the integrase gene, intI2, of class II integrons encodes 325 amino acids, which share 46% homology with intI1 (19); the integrase gene intI3 of class III integrons encodes 320 amino acids, having 61% homology with intI1 (20). In view of the homology that the integrases of the three classes of integrons have (21), the multiplex PCR method was used to amplify these three types of integrases. Primers for these three types of integrases, consistent with the multiplex PCR amplification conditions, were designed based on their shared sequences. The designed primers of class I, II and III integrons have similar annealing temperatures, significantly different product sizes and the electrophoresis bands are easily differentiated. The experimental results showed that the designed integrase primers of class I, II and III integrons are capable of effectively detecting and differentiating the class I, II and III integrons using the multiplex PCR method. Integrons, which exist in the genomic DNA and plasmid DNA, can be disseminated between the same and different bacterial genera through transposons, integrating bacteriophages and conjugal plasmids. These experimental results indicated that the positive rate of class I and II integrons in SA plasmid DNA was higher than that in the SA genomic DNA. The reason for this may be that the drug resistance gene of class II integrons in plasmids can easily take part in horizontal transfer.

At present, the majority of box genes found in class I integrons are drug resistance box genes, whose encoding product enables bacteria to resist almost all the antibiotics applied in the clinic. One integron may carry several box genes. The production of bacterial multidrug resistance is closely related to the integrons. Integrons are commonly observed in Gram-negative bacteria (22). The experimental subject in this study was SA, a Gram-negative bacteria. Research in China on SA is comparatively rare. The experimental results indicated that the class I integron detection rate was highest in the strains resistant to most types of antibiotics, which was identical to the findings of Madiyarov *et al*(23). This indicated that the detection rate of class I integrons positively correlated with the SA drug resistance. This finding proposes a new direction for the research of bacterial multi-drug resistance mechanisms. The drug resistance gene may be transferred between different integrons through integrating or removing the box gene under the influence of integrase, and disseminated through the movement of plasmids and transposons (13). Therefore, the fast experimental detection of integrons may be regarded as a useful tool for the monitoring of bacterial drug resistance.

In this experiment, after comparing the class I and II integron detection conditions of SA specimens from sputum, blood, cerebrospinal fluid, drain fluid, excretion and urine, the class I integron detection rate in the plasmid DNA from the urine specimen was the highest, followed by plasmid DNA from the sputum specimen. The reason for the comparatively higher amount of integrons in SA obtained from the urine specimens (24) may be that infection found in the urine had already been serious when bacteria invaded the urine, and the environment that drug-resistant SA was in had positive selection. The class I integron positive rate of SA from the urine specimen was higher than that from other specimens, which indicated that the SA drug resistant environment had a certain influence on the carrying rate of integrons.

In conclusion, multiplex PCR is an effective method to detect class I, II and III integrons, which has the advantage of saving time and resources. The carrying rate of class I and II integrons of in plasmid DNA was higher than that in genomic DNA in drug-resistant SA, which indicated that the plasmid is the main carrier for transferring integron. The more drug-resistant the SA, the higher the carrying rate of class I integron, which indicated that integron mediates the SA multidrug resistance. SA plasmid extracted from the specimen with a high rate of drug resistance had a high class I integron carrying rate, which indicated that the SA environment had a certain influence on the carriage of integrons.

## Figures and Tables

**Table I:** Drug resistance in *Staphylococcus aureus* (SA).

Drugs	Number of resistant strains (n=180)	Drug resistance rate (%)
Cefoxitin	95	52.8
Benzylpenicillin	169	93.7
Oxacillin	94	52.2
Erythromycin	146	81.1
Azithromycin	143	79.4
Clindamycin	123	68.3
Vancomycin	0	0.0
Bactrim	87	48.3
Linezolid	5	2.8
Amoxicillin/clavulanic acid	102	56.7
Piperacillin/tazobactam	99	55.0
Ciprofloxacin	103	57.2
Tetracycline	102	56.7
Rifampicin	75	41.7
Imipenem	91	50.6
Cefazolin	93	51.7
Cefuroxime	97	53.9
Levofloxacin	36	19.8
Gentamicin	94	52.2
Teicoplanin	4	2.2

**Table II:** Comparison of class I integron detection in *Staphylococcus aureus* (SA) plasmid and genomic DNA.

	Class I integrons		
		
DNA resources	Positive strains	Negative strains	Positive rate (%)
Genomic DNA	71	109	39.44
Plasmid DNA	92	88	51.11
χ^2^		4.94	
P-value		0.026	

**Table III:** Comparison of class II integron detection in *Staphylo- coccus aureus* (SA) plasmid and genomic DNA.

	Class II integrons		
		
DNA resources	Positive strains	Negative strains	Positive rate (%)
Genomic DNA	10	170	5.88
Plasmid DNA	23	157	12.78
χ^2^		5.64	
P-value		0.018	

**Table IV:** Class I and II integron detection in *Staphylococcus aureus* (SA) plasmid DNA with different SA drug resistance.

Drug-resistant type	Number of resistant strains	Positive rate of class I integron (%)	Positive rate of class II integron (%)
A	80	43.8	8.8
B	62	59.7	16.1
C	25	80.0	24.0
χ^2^		9.28	4.19
P-value		<0.001	0.15

A, group with drug-resistant strains that resist penicillin, cefoxitin, or oxacillin; B, group with drug-resistant strains that resist erythromycin, azithromycin, trimethoprim-sulfamethoxazole, clarithromycin, clindamycin and linezolid; C, group with drug-resistant strains that resist amoxicillin/clavulanic acid, piperacillin/tazobactam, ciprofloxacin, tetracycline, rifampin, imipenem, cefazolin, cefuroxime, levofloxacin and gentamicin.

**Table V:** Detection of class I integrons in the *Staphylococcus aureus* (SA) plasmid DNA from different specimen sources.

Strain source	Multidrug resistant rate (%)	Class I integrons		Positive rate (%)

		Positive strains	Negative strains	
Sputum	67.9	52	34	60.47
Blood	28.3	4	22	15.38
Drain	42.9	7	8	46.67
Excretion	39.7	5	13	27.78
Cerebrospinal fluid	66.7	1	2	33.33
Urine	79.4	21	11	65.63
Total		90	90	-

**Table VI:** Detection of class II integrons in the *Staphylococcus aureus* (SA) plasmid DNA from different specimen sources.

Strain source	Multidrug resistant rate (%)	Class II integrons		Positive rate (%)

		Positive strains	Negative strains	
Sputum	67.9	11	75	12.79
Blood	28.3	1	25	3.85
Drain	42.9	2	13	13.33
Excretion	39.7	1	17	5.56
Cerebrospinal fluid	66.7	0	3	0
Urine	79.4	8	24	25.0
Total		23	157	-
